# FGF-trapping hampers cancer stem-like cells in uveal melanoma

**DOI:** 10.1186/s12935-023-02903-z

**Published:** 2023-05-11

**Authors:** Alessandra Loda, Stefano Calza, Arianna Giacomini, Cosetta Ravelli, Adwaid Manu Krishna Chandran, Chiara Tobia, Giovanna Tabellini, Silvia Parolini, Francesco Semeraro, Roberto Ronca, Sara Rezzola

**Affiliations:** 1 Department of Molecular and Translational Medicine, University of Brescia, viale Europa 11, 25123 Brescia, Italy; 2 Eye Clinic, Department of Medical and Surgical Specialties, Radiological Sciences and Public Health, University of Brescia, Brescia, Italy

**Keywords:** Uveal melanoma, Cancer stem-like cells, FGF, FGF inhibitor

## Abstract

**Background:**

Cancer stem-like cells (CSCs) are a subpopulation of tumor cells responsible for tumor initiation, metastasis, chemoresistance, and relapse. Recently, CSCs have been identified in Uveal Melanoma (UM), which represents the most common primary tumor of the eye. UM is highly resistant to systemic chemotherapy and effective therapies aimed at improving overall survival of patients are eagerly required.

**Methods:**

Herein, taking advantage from a pan Fibroblast Growth Factor (FGF)-trap molecule, we singled out and analyzed a UM-CSC subset with marked stem-like properties. A hierarchical clustering of gene expression data publicly available on The Cancer Genome Atlas (TCGA) was performed to identify patients’ clusters.

**Results:**

By disrupting the FGF/FGF receptor (FGFR)-mediated signaling, we unmasked an FGF-sensitive UM population characterized by increased expression of numerous stemness-related transcription factors, enhanced aldehyde dehydrogenase (ALDH) activity, and tumor-sphere formation capacity. Moreover, FGF inhibition deeply affected UM-CSC survival in vivo in a chorioallantoic membrane (CAM) tumor graft assay, resulting in the reduction of tumor growth. At clinical level, hierarchical clustering of TCGA gene expression data revealed a strong correlation between FGFs/FGFRs and stemness-related genes, allowing the identification of three distinct clusters characterized by different clinical outcomes.

**Conclusions:**

Our findings support the evidence that the FGF/FGFR axis represents a master regulator of cancer stemness in primary UM tumors and point to anti-FGF treatments as a novel therapeutic strategy to hit the CSC component in UM.

**Supplementary Information:**

The online version contains supplementary material available at 10.1186/s12935-023-02903-z.

## Highlights

- Overexpression of FGFs/FGFRs correlates with stemness and fate in UM patients
- Blockade of FGFs unmasks an FGF-sensitive UM population with marked stem-like properties
- FGF trapping inhibits the growth of UM by targeting cancer stem cells

**Supplementary Information:**

The online version contains supplementary material available at 10.1186/s12935-023-02903-z.

## Background

Uveal melanoma (UM) is the most common primary intraocular malignancy, arising from melanocytes located in the uveal tract of the eye [1]. Incidence of UM in Europe ranges from 2 to 8 per million and its occurrence increases with age [2]. Commonly, primary tumors are successfully treated with brachytherapy and phototherapy, while enucleation remains an appropriate procedure in the presence of large tumors with extensive extraocular growth [3]. However, despite effective control of localized tumors, UM is very aggressive, and it tends to spread via hematological dissemination [4]; more than 50% of patients develop metastasis, most frequently to the liver, with median survival after diagnosis ranging from 3 to 12 months [4, 5]. UM is highly resistant to systemic chemotherapy and no standard of care has been approved for treatment of metastatic disease [2, 6, 7]. Moreover, the low mutational burden of UM, the immunoprivileged site of the eye, as well as the immunosuppressive environment of the liver hamper the efficacy of novel approaches based on immunotherapy [8, 9]. Therefore, effective therapies aimed to improve overall survival of patients are currently lacking [8, 10]. In this context, experimental models of UM represent a useful tool for the identification of new potential drugs [11].

Fibroblast growth factors (FGFs) are involved in several physiological processes such as embryogenesis, angiogenesis, tissue homeostasis, and wound repair, by acting as paracrine, autocrine, or endocrine factors which activate tyrosine-kinase FGF receptors (FGFRs) [12, 13]. The aberrant activation of the FGF/FGFR system is frequently observed in human cancers, affecting cell proliferation, differentiation, migration, and survival [13]. The constitutive activation of the FGF/FGFR system has been described in UM, where the overexpression of the ligands and/or receptors promotes an autologous loop of stimulation which sustains UM progression [14–17]. Recently, we have identified the novel small molecule NSC12 as a pan-FGF-trap able to bind to FGFs and prevent FGFR activation. By disrupting FGF/FGFR-mediated signaling, NSC12 has been shown to hamper tumor growth in several FGF-dependent murine and human cancer models, including UM [18–21]. Indeed, primary and metastatic UM cell lines showed impaired cell migration, proliferation, and survival after treatment with NSC12 [21].

Cancer Stem-like Cells (CSCs) represent a subpopulation of cells responsible for tumor initiation, growth, and metastasis [22–24]. Moreover, CSCs are resistant to both chemotherapy and radiotherapy due to several mechanisms, including their lower proliferation rate, the activation of the DNA repair machinery, and the expression of transporters and enzymes that internalize and inactivate drugs [25, 26]. The presence of CSCs has been reported in various tumor types, such as cutaneous melanoma, breast, lung, liver, stomach, and bladder cancers [22]. In this context, FGFs reportedly contribute to pluripotency maintenance and self-renewal of stem cells both in normal tissues and in several tumor types [27–31]. Markers of CSCs vary according to the type of cancers, and may include transcriptional factors (*e.g.* NANOG, OCT4, SOX2), as well as surface proteins such as CD44, CD133, and CD47 [32, 33]. At present, due to their role in promoting tumor heterogeneity, resistance to therapies and recurrence, targeting CSCs with new therapeutic approaches represents a first line challenge to obtain complete tumor eradication [34].

Recently, CSCs have also been identified in UM as a subgroup of cells characterized by increased motility, self-renewal, and chemoresistance [35–37]. Given the absence of reliable surface markers for CSCs in UM [38], current studies assessed their presence by evaluating stem-like properties such as formation of melanospheres and enhanced activity of aldehyde dehydrogenase (ALDH) enzymes [37, 39–42].

In this paper, we demonstrate that sequestration of FGFs by NSC12 hits and unmasks an FGF-sensitive UM population with marked stem-like properties. By targeting the UM-CSC subpopulation, blockade of FGFs results in the inhibition of UM growth both in vitro and in the chick embryo chorioallantoic membrane (CAM) in vivo model. Moreover, we show that FGF/FGFR expression and stemness are strictly linked in UM patients and are associated with poorer prognosis. Altogether, these findings indicate that the FGF system plays a pivotal role in UM-CSC biology and may be exploited to develop novel anti-CSC strategies for UM.

## Methods

### Reagents

RPMI 1640 medium, fetal bovine serum (FBS), and SYBR Green PCR master mix were from GIBCO Life Technologies (Grand Island, NY, USA). Penicillin, streptomycin, Triton-X100, BriJ, sodium orthovanadate, protease inhibitor cocktail, bovine serum albumin (BSA), and 4′,6-diamidino-2-phenylindole (DAPI) were from Sigma-Aldrich (St. Louis, MO, USA). Bradford reagent, enhanced chemiluminescence reagent, and iTaq Universal Syber Green Supermix were from Bio-Rad Laboratories (Hercules, CA, USA). TRIzol Reagent, Moloney murine leukemia virus (MMLV) reverse transcriptase, and MitoSox Red Mithocondrial Superoxide Indicator were from Invitrogen (Carlsbad, CA, USA). 2X XtraRTL Master Mix was from GeneSpin (Milan, Italy). ALDEFLUOR kit was from Stemcell Technologies (Vancouver, Canada). Human Phospho-Kinase Array Kit was from R&D Systems (Minneapolis, Canada). Anti-phospho-pan-FGFR (Tyr653/Tyr654), anti-Nanog, and anti-phospho-paxillin (Tyr118) antibodies were from Cell Signaling Technology (Beverly, MA, USA). Anti-GAPDH and anti-FGFR1 (C-15) antibodies were from Santa Cruz (Santa Cruz, CA, USA). Chicken anti-rabbit Alexa Fluor 488 and phalloidin-Alexa Fluor 594 antibodies were from Molecular Probes (Eugene, OR, USA). Recombinant FGF2 was purchased from Tecnogen (Caserta, Italy). NSC12 was kindly provided by Dr. M. Mor (University of Parma, Italy).

### Cell cultures

Human UM cell lines 92.1, Mel285, and Mel270 were obtained from M. Jager (Leiden University, The Netherlands) and cultured in RPMI 1640 medium supplemented with 100 U/mL penicillin, 100 µg/ml streptomycin, and 10% FBS or 20% FBS for 92.1 and Mel285 or Mel270 UM cells, respectively [43–45].

When required, 92.1 and Mel270 cells were seeded at 13,000 cells/cm^2^ in complete medium and then starved in RPMI plus 1% FBS. After 24 h, cells were treated with 15 µM NSC12. NSC12^sens^ population was identified as the fraction that detached from the substratum after either 2 h or 3 h of treatment for 92.1 and Mel270 cells, respectively, whereas NSC12^res^ population remained adherent to the cell culture plastic.

### Western blot analysis

NSC12^sens^ and NSC12^res^ cells were harvested, and samples were homogenized in RIPA buffer containing 1% Triton-X100, 0.2% BriJ, 1 mM sodium orthovanadate, and protease inhibitor cocktail. Total lysates (50 µg) cells were separated by SDS-PAGE and probed with specific antibodies. Western blot analysis was performed using rabbit anti-pan-phospho-FGFRs, rabbit anti-FGFR1, rabbit anti-Nanog and rabbit anti-phospho-Paxillin antibodies and normalized using mouse anti-GAPDH antibody. Primary antibodies were diluted 1:1000, while secondary antibodies were diluted 1:5000 in blocking solution (TBS 1% Tween 20 supplemented with 1% BSA). Chemiluminescent signal was acquired by ChemiDoc^™^ Imaging System (Bio-Rad) and quantified by Fiji software [46].

### Human phospho-protein proteome profiler array

NSC12^sens^ and NSC12^res^ 92.1 cell lysates were analyzed to assess the phosphorylation levels of several intracellular proteins using the proteome profiler array Human Phospho-Kinase Array Kit (R&D Systems, Minneapolis, Canada). 500 µg of total lysates of NSC12^sens^ and NSC12^res^ 92.1 cells were incubated with the array according to manufacturer’s instructions. Pixel densities were analyzed using the image analysis Fiji software and normalized on reference spots.

### Immunofluorescence analysis

92.1 and Mel270 cells were seeded 15,000 cells/cm^2^ in RPMI 10% or 20% FBS, respectively. After 24 h, cells were treated with 15 µM NSC12 for 1 h, 2 h, or 3 h in RPMI 1% FBS. Then, cells were washed, fixed with 4% paraformaldehyde and permeabilized using PBS 0.2% Triton-X100. Blocking was performed using PBS 0.1% Tween20 supplemented with 1% bovine serum albumin (blocking solution). Primary rabbit anti-phospho-paxillin antibody was diluted 1:100 in blocking solution and cells were incubated for 1 h at room temperature. Then, cells were incubated for 1 more h at room temperature with anti-rabbit secondary antibody (diluted 1:250 in blocking solution) along with phalloidin-Alexa Fluor 594 (diluted 1:150 in blocking solution). Lastly, nuclei were counterstained with DAPI diluted 1:15000 in PBS. Cells were photographed using an Axiovert 200 M epifluorescence microscope equipped with Apotome and a Plan-Apochromat × 63/1.4 NA oil objective (Zeiss). Image analysis was performed using Fiji software.

### Semi-quantitative PCR analysis

Control, NSC12^sens^ and NSC12^res^ cells were harvested, and total RNA was extracted using Trizol Reagent. Contaminating DNA was eliminated using DNAse before performing retrotranscription. For each sample, 2 µg of RNA were retrotranscribed using MMLV reverse transcriptase. cDNA was then amplified using the oligonucleotide primers listed in Additional file 1: Table SI. The PCR products were electrophoresed on a 2.5% agarose gel.

### Quantitative real-time PCR (qPCR) analysis

NSC12^sens^ and NSC12^res^ cells were harvested, and total RNA was extracted and retrotranscribed. cDNA was analyzed using the iTaq Universal Syber Green Supermix with the ViiA7 Real-Time PCR System. Samples were analyzed in triplicate using the oligonucleotide primers reported in Additional file 1: Table SII.

### Cytofluorimetric analysis

Control, NSC12^sens^ and NSC12^res^ cells were harvested and analyzed by cytofluorimetry. Mitochondrial reactive oxygen species (mtROS) production was determined using the fluorescent probe MitoSox; apoptotic cell death was evaluated by double staining with Annexin-V/Propidium Iodide; ALDH activity was assessed using ALDEFLUOR kit. Staining for *NANOG* was performed with anti-NANOG antibody conjugated with Alexa Fluor 647. Each assay was performed according to the manufacturer’s instructions. Cytofluorimetric analyses were performed using the MACSQuant^®^ Analyzer (Miltenyi Biotec, Bergisch Gladbach, Germany).

### Tumor-sphere assay

92.1, Mel270, and Mel285 cells were pre-treated with increasing doses of NSC12 for 24 h. Then, 3000 viable cells were seeded on low-adhesion plates (Corning) in DMEM/F12 medium supplemented with 100 U/mL penicillin, 100 µg/ml streptomycin, 10 ng/ml FGF2, 20 ng/ml EGF, and B-27 supplement (diluted 1:50). After 7 days, spheres were counted.

### Chick embryo chorioallantoic membrane (CAM) assay

Fertilized white Leghorn eggs were incubated at 37 °C in a humidified incubator. At 4 days post-incubation, the shells were covered with a transparent adhesive tape and a small window cut with scissors; windows were then resealed with tape. At 7 days post-incubation, 92.1 cell were engrafted on the CAM at a concentration of 100,000 cells/µl in 1:1 Matrigel/PBS (*vol: vol*). NSC12 was added into the cell suspension directly before engraftment (4 pmol/embryo). At 14 days post-incubation tumors were photographed and then explanted. Tumor volume was calculated using the following formula: V = (D x d^2^)/2, where D and d are the major and minor perpendicular tumor diameters, respectively [47]. RNA was extracted from the grafts and gene expression was analyzed by Real Time PCR.

### Statistical analyses

Independent groups were compared using one-way analysis of variance followed by pairwise comparisons with Tukey HSD p-value adjustment.

Data were clustered using hierarchical clustering with Euclidean distance metric and Ward’s agglomerative clustering method [48]. Best number of clusters was determined using the consensus approach provided by the NbClust package [49], *i.e.* as the optimal number of clusters most commonly selected out of 30 different indices. Geneset (FGFs, FGFRs, Stemness) overall expression was computed using single-sample gene set enrichment analysis as provided by gene set variation analysis algorithm [50]. Briefly, the overall expression of a collection of genes (geneset) is computed using a non-parametric model that map from a multiple expression space (gene expression values) to a single cumulative expression value for the gene set. Survival curves relative to patients Disease-Free Survival (DFS) were plotted using Kaplan–Meier estimator and *p*-value computed using a log-rank test.

All tests were two-sided and assumed a 5% significance level. All statistical analyses were performed with GraphPad Prism 9 (San Diego, CA, USA) and R (version 4.0.2).

## Results

### FGF-trapping identifies a subpopulation of UM cells highly dependent on FGF signaling

**Fig. 1:**
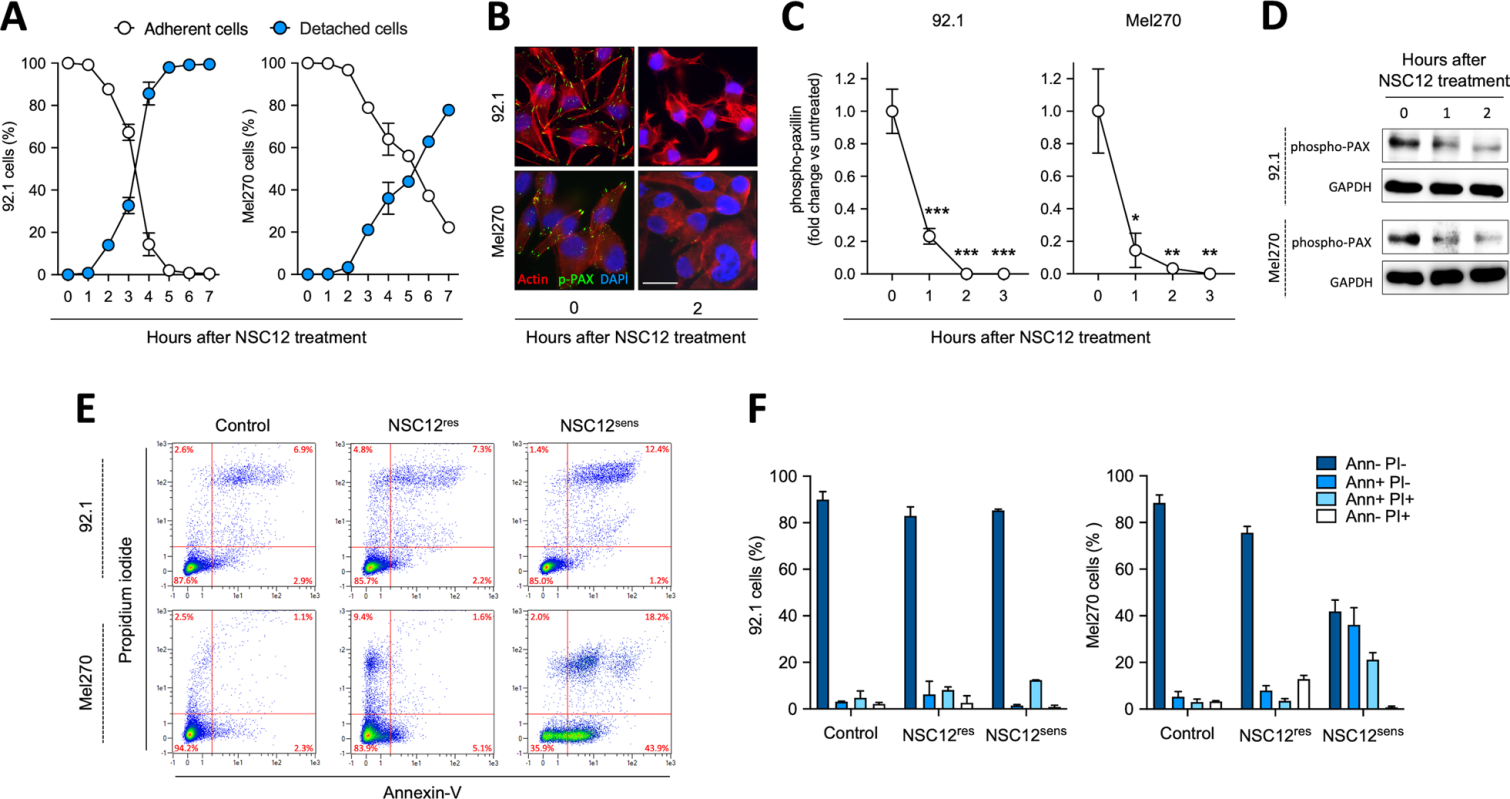
Treatment with NSC12 identifies an FGF-dependent subpopulation in UM cells. **A** 92.1 and Mel270 cells were treated with 15 µM NSC12 and its effect on cell adhesion was followed over time. Every hour adherent and non-adherent cells were collected and counted. **B** Immunofluorescence analysis of actin (red fluorescence) and phospho-paxillin (p-PAX, green fluorescence) expression in 92.1 (upper panels) or Mel270 (lower panels) cells treated or not with 15 µM NSC12 for 2 h. Nuclei were counterstained with DAPI (blue fluorescence). Scale bar = 30 µm. **C** Quantification of phospho-paxillin fluorescence signal was normalized to the number of nuclei. Data are the mean ± SEM of 10 fields (n = 70 cells). *p < 0.05, **p < 0.01, ***p < 0.001 vs control, ANOVA. **D** Western blot analysis of phospho-PAX in control and NSC12-treated 92.1 (upper panels) and Mel270 (lower panels) cells. Data are representative of two independent experiments that gave similar results (see Additional file 1: Fig. S1). **E** 92.1 and Mel270 cells were treated or not with 15 µM NSC12 for 2 h (92.1) or 3 h (Mel270). Then, control, NSC12^sens^ and NSC12^res^ cells were harvested, and apoptosis was analyzed by cytofluorimetry. **F** Apoptosis quantification of propidium iodide (PI) and Annexin-V (Ann) positive cells by MACSQuant Software. Data are the mean ± SEM of three independent experiments

Previous observations had shown that the pan-FGF trap NSC12 inhibits FGFR activation and its downstream signaling both in primary and metastatic human UM cell lines. Additionally, NSC12 promotes the activation of the pro-apoptotic proteins PARP and caspase-3, thus leading to UM cell death [21]. Here, human 92.1 and Mel270 UM cells were seeded at subconfluent density and allowed to adhere to the tissue culture plastic. Then, cells were treated with NSC12 and their behavior was followed thereafter. As shown in Fig. 1A, during the first few hours of treatment (*i.e.* 2 and 3 h for 92.1 and Mel270 UM cells, respectively) a small percentage of cells (≃ 20% of the total) detached from the tissue culture plastic. Notably, the detachment phenotype was rescued by the addition of a 1:1 molar concentration ratio of FGF2 (Additional file 1: Fig. S1). In addition, treatment with NSC12 induced morphological modifications of the cytoskeleton, with alterations of actin organization and significant dephosphorylation of paxillin at focal adhesions (Fig. 1B–D and Additional file 1: Fig. S2). This approach allowed to identify and isolate a cell population adherent to the substratum and resistant to FGF deprivation (NSC12^res^) and a non-adherent cell population sensitive to the treatment with the anti-FGF drug (NSC12^sens^). Of note, the evaluation of the apoptotic rate of NSC12^res^/NSC12^sens^ populations confirmed their viability in both UM cell lines, even though 35% of Mel270_NSC12^sens^ cells showed sign of early apoptosis with positive staining for Annexin-V (Fig. 1E, F).

**Fig. 2:**
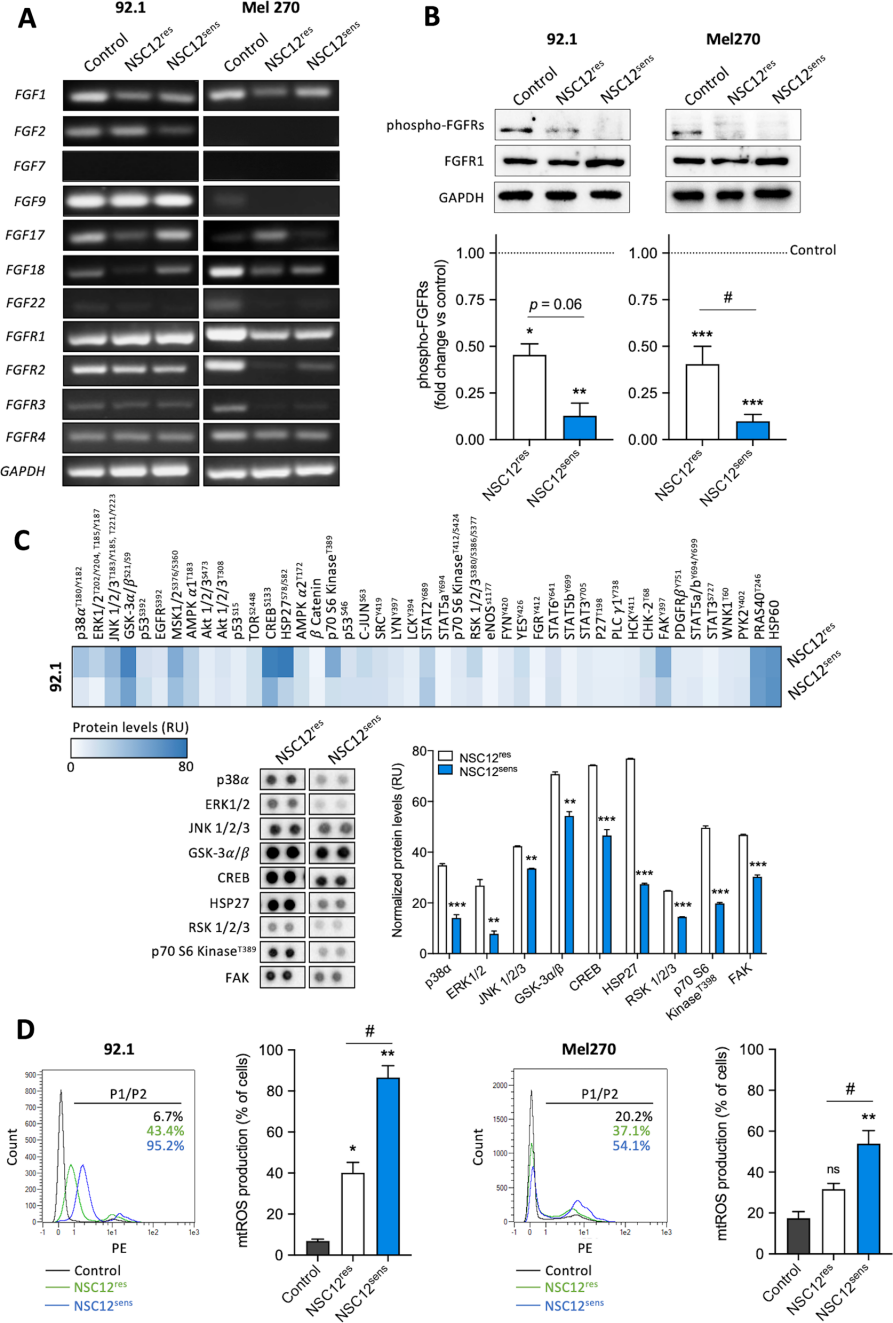
Characterization of NSC12^res^/NSC12^sens^ UM subpopulations. **A** Semi-quantitative PCR analysis of FGF and FGFR expression in control and NSC12^res^/NSC12^sens^ subpopulations of 92.1 (left panels) and Mel270 (right panels) cells. **B** Western blot analysis of phospho-FGFRs and FGFR1 in control and NSC12^res^/NSC12^sens^ subpopulations of 92.1 (left panels) and Mel270 (right panels) cells. The lower panels show the densitometric analysis of immunoreactive bands normalized to GAPDH protein levels. Data are the mean ± SEM of three independent experiments. *p < 0.05 and **p < 0.01 vs control; ^#^*p* < 0.05 vs NSC12^res^, ANOVA. **C** Human Phospho-Kinase Antibody Array on 92.1_NSC12^res^ and 92.1_NSC12^sens^ total cell lysates. The heatmap shows the color-coded normalized protein levels of all detected phospho-proteins. The lower panels show the spots of p38∝, ERK1/2, JNK1/2/3, GSK-3∝/β, CREB, HSP27, RSK 1/2/3, p70 S6 Kinase and FAK and the corresponding densitometric analysis (relative units, RU). Data are the mean ± SEM of two technical replicates in one representative experiment out of three independent measurements that provided similar results. **p < 0.01 and ***p < 0.001 vs NSC12^res^, Student’s *t*-test. **D** The production of mtROS was assessed on control and NSC12^res^/NSC12^sens^ subpopulations of 92.1 (left panels) and Mel270 (right panels) cells using the fluorescent probe MitoSox by cytofluorimetric analysis. In the plots the black line refers to the gate (P1/P2) setting the positive/negative cell populations. Data are the mean ± SEM of three independent experiments. *p < 0.05 and **p < 0.01 vs control; ^#^*p* < 0.05 vs NSC12^res^, ANOVA

UM primary tumors express several FGFs and FGFRs [21]. To assess if the higher sensitivity of NSC12^sens^ cells to FGF deprivation was due to different FGF/FGFR expression levels, NSC12^res^ and NSC12^sens^ populations obtained from 92.1 and Mel270 cells were analyzed by semi-quantitative PCR. As shown in Fig. 2A, both NSC12^res^ and NSC12^sens^ cells express similar levels of FGFs and FGFRs; moreover, treatment with NSC12 triggered a significant inhibition of FGFR phosphorylation in both populations (Fig. 2B).

To get further insights on the impact of FGF blockade in UM, we performed a Phospho-Kinase Antibody Array analysis of NSC12^res^/NSC12^sens^ 92.1 cells. As shown in Fig. 2C, NSC12^sens^ cells displayed decreased phosphorylation of various intracellular kinases when compared to NSC12^res^ cells. NSC12^sens^ population showed lower levels of phospho-FAK and a downregulation of p38 signaling pathway with decreased levels of phospho-p38 and its targets phospho-CREB and phospho-HSP27. In addition, when compared to NSC12^res^ cells, JNK and ERK MAPK signaling pathways were turned off, as demonstrated by reduced phospho-JNK1/2/3, phospho-ERK1/2, phospho-RSK1/2/3, phospho-GSK-3α/β and phospho-p70 S6 kinase levels.

In keeping with previous observations on FGF/FGFR inhibition and intracellular oxidative stress induction [20, 51], NSC12^sens^ cells showed an increased production of mtROS compared to untreated and NSC12^res^ cells, revealing a strong mitochondrial oxidative stress response in this cell population following treatment (Fig. 2D).

Together, these data demonstrate that in UM exists a subpopulation of cells that, in response to FGF-trapping, shows an earlier and stronger mitochondrial stress response as well as a down-modulation of several pro-survival mediators.

### NSC12 inhibits UM growth UM growth in vitro and in vivo by targeting the UM-CSC subpopulation

**Fig. 3:**
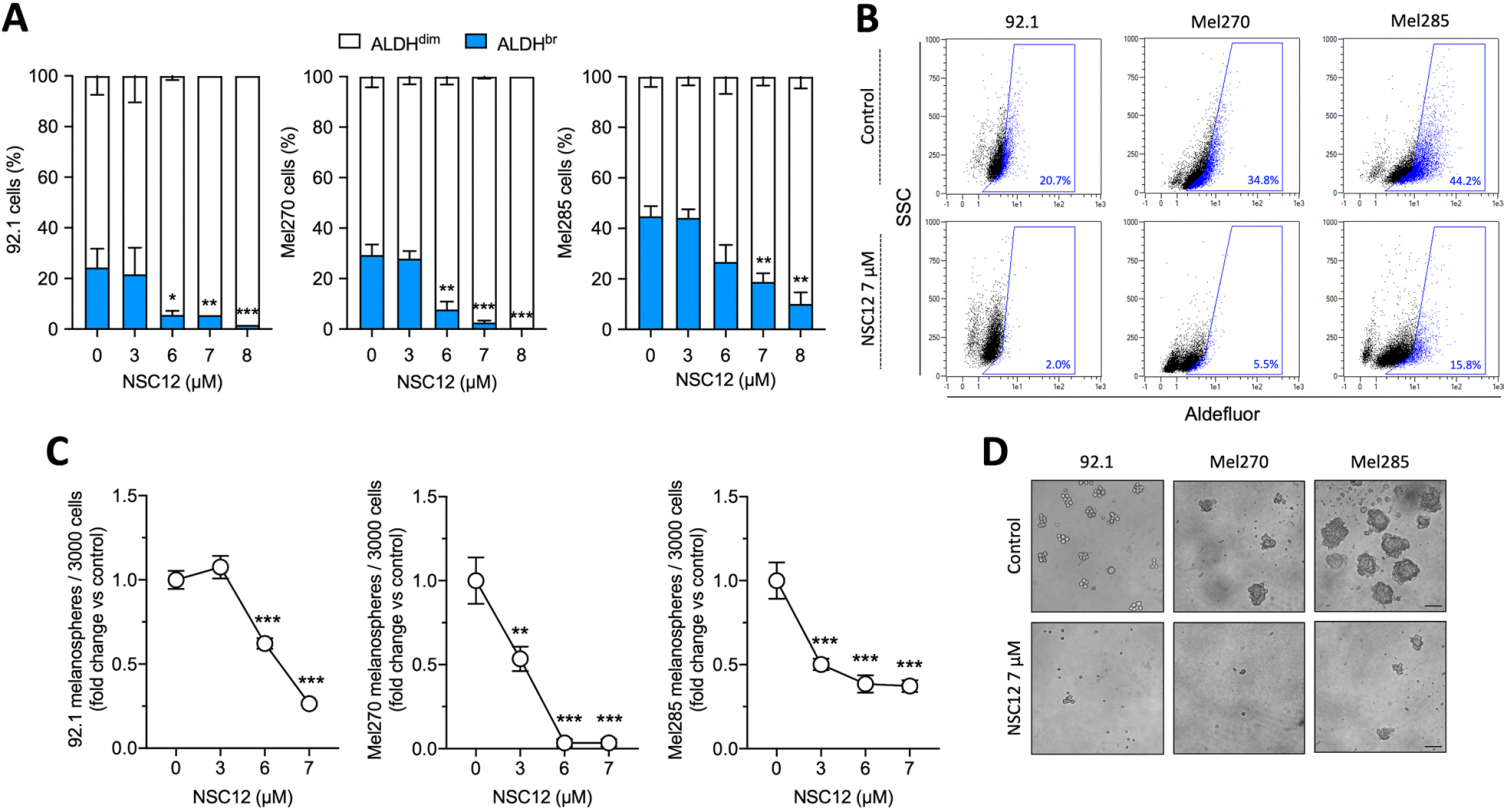
Treatment with NSC12 reduces the self-renewal potential of UM cells. **A** 92.1, Mel270 and Mel285 cells were treated with increasing doses of NSC12 for 24 h. Then, ALDH^br^ cells were measured by cytofluorimetric analysis. Data are the mean ± SEM of three independent experiments. *p < 0.05, **p < 0.01, ***p < 0.001 vs control, ANOVA. **B** Representative flow cytometry dot plots of control or 7 µM NSC12 treated UM cells. The blue gate refers to the positive/negative cell populations as identified in the presence of DEAB inhibitor (see Additional file 1: Fig. S2). **C** 92.1, Mel270 and Mel285 cells were treated with increasing doses of NSC12 for 24 h. Then 3000 viable cells were resuspended in melanospheres culture medium and plated. After 7 days, melanospheres were counted and photographed. Data are the mean ± SEM of three independent experiments. **p < 0.01, ***p < 0.001 vs control, ANOVA. **D** Representative images of melanospheres obtained from control or 7 µM NSC12 pre-treated UM cells. Scale bar: 100 µm

A CSC subpopulation has been identified in UM cell lines based on the ability to form melanospheres and the activity of ALDH enzymes [37, 39–42]. By evaluating these properties, we identified a UM-CSC subpopulation, confirming the presence of CSCs in human 92.1, Mel270, and Mel285 UM cell lines (Additional file 1: Fig. S3). The FGF/FGFR system plays a pivotal role in stem cell maintenance [27–31]; given the presence of distinct subpopulations with different sensitivity to FGF deprivation, we investigated the effect of NSC12-mediated FGF blockade on the UM-CSC subset. As shown in Fig. 3A, B and Additional file 1: Fig. S4, treatment with increasing doses of NSC12 resulted in a significant reduction of the ALDH^br^ population in 92.1, Mel270, and Mel285 UM cells. Similar results were obtained after treatment with the FGFR-selective tyrosine-kinase inhibitor BGJ398, thus confirming the FGF/FGFR-restricted specificity of this effect (Additional file 1: Fig. S5). Interestingly, UM-ALDH^br^ cells were resistant to the treatment with standard chemotherapy drug dacarbazine, in accordance with their stem-like traits (Additional file 1: Fig. S6). Additionally, in keeping with ALDH enzymatic activity data, pretreatment with NSC12 resulted in a dose-dependent decrease of the number of melanospheres (Fig. 3C, D), confirming that FGF signaling plays a key role in maintaining stem-like features of UM cells in vitro and that FGF sequestration pauperizes the CSC subpopulation in UM cells.

**Fig. 4:**
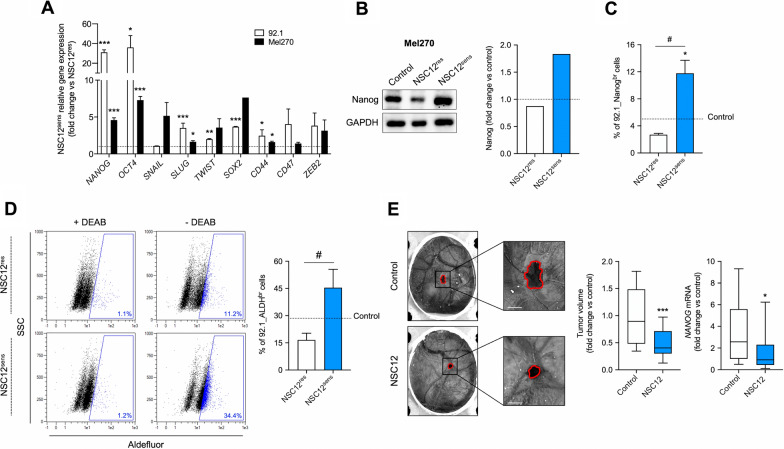
NSC12^sens^ cells are characterized by stem-like features. **A** qPCR analysis of *NANOG*, *OCT4*, *SNAIL*, *SLUG*, *TWIST*, *SOX2*, *CD44*, *CD47*, and *ZEB2* expression in NSC12^res^ and NSC12^sens^ subpopulations of 92.1 and Mel270 cells. Data are the mean ± SEM of three independent experiments. *p < 0.05, **p < 0.01, ***p < 0.001 vs NSC12^res^, Student’s *t*-test. **B** Western blot analysis of Nanog in control and NSC12^res^/NSC12^sens^ Mel270 cells. Right panel: densitometric analysis of immunoreactive bands normalized to GAPDH protein levels. **C** Nanog^br^ cells were measured on control and NSC12^res^/NSC12^sens^ 92.1 cells by cytofluorimetric analysis. Data are the mean ± SEM of three independent experiments. *p < 0.05 vs control; ^#^*p* < 0.05 vs NSC12^res^, ANOVA. **D** ALDH^br^ cells were measured on control and NSC12^res^/NSC12^sens^ 92.1 cells by cytofluorimetric analysis. Representative dot plots are reported in the left panel. Data are the mean ± SEM of three independent experiments. ^#^*p* < 0.05 vs NSC12^res^, Student’s *t*-test. **E** 92.1 cells were engrafted onto the chick embryo CAM at day 7 post-incubation in the absence or in the presence of 4 pmol/embryo NSC12. Tumor growth was assessed after 7 days and qPCR analysis of *NANOG* was performed on the explants (right panels). In box and whisker graphs, boxes extend from the 25th to the 75th percentiles, lines indicate the median values, and whiskers indicate the range of values. Data are the mean ± SEM of two independent experiments (n = 20). *p < 0.05, ***p < 0.001 vs control, Student’s *t*-test. Representative images of tumors are shown in the left panel. Scale bar: 2 mm

Based on these results and to further characterize the stem-like features of the NSC12^sens^ population, we analyzed the expression levels of a panel of transcription factors known to be involved in stemness maintenance. To this purpose, NSC12^res^ and NSC12^sens^ cells obtained from 92.1 and Mel270 cells were evaluated by qPCR. As shown in Fig. 4A, when compared to NSC12^res^ cells, the NSC12^sens^ population expressed higher levels of several markers of stemness, including *SOX2*, *SLUG*, *TWIST*, *OCT4* and *NANOG*. Accordingly, Mel270_NSC12^sens^ cells expressed more Nanog when analyzed by Western blotting (Fig. 4B). In line with these results, cytofluorimetric analysis further confirmed that 92.1_NSC12^sens^ cells were enriched in Nanog^br^ cells and were endowed with the highest ALDH enzymatic activity (Fig. 4C, D).

Finally, to evaluate whether NSC12 could affect the CSC population and impair tumor growth of UM cells in vivo, 92.1 cells were engrafted on the chick embryo CAM at day 7 post-incubation and treated with 4 pmol/embryo of NSC12. Tumor growth was assessed 7 days post-implantation and grafts were explanted and analyzed by qPCR. As shown in Fig. 4E, tumor mass was significantly reduced after treatment with NSC12 compared to controls. In addition, tumors treated with NSC12 were characterized by a strong downregulation of *NANOG* expression as evaluated by qPCR, thus confirming the reduction of the CSC component in the NSC12-treated group. Altogether, these data suggest that UM-CSCs strictly depend on FGF signaling and that FGF/FGFR axis inhibition may result in a strong effect on the UM-CSC compartment in vitro and in vivo.

### Overexpression of FGFs/FGFRs correlates with stemness and fate in UM patients

**Fig. 5:**
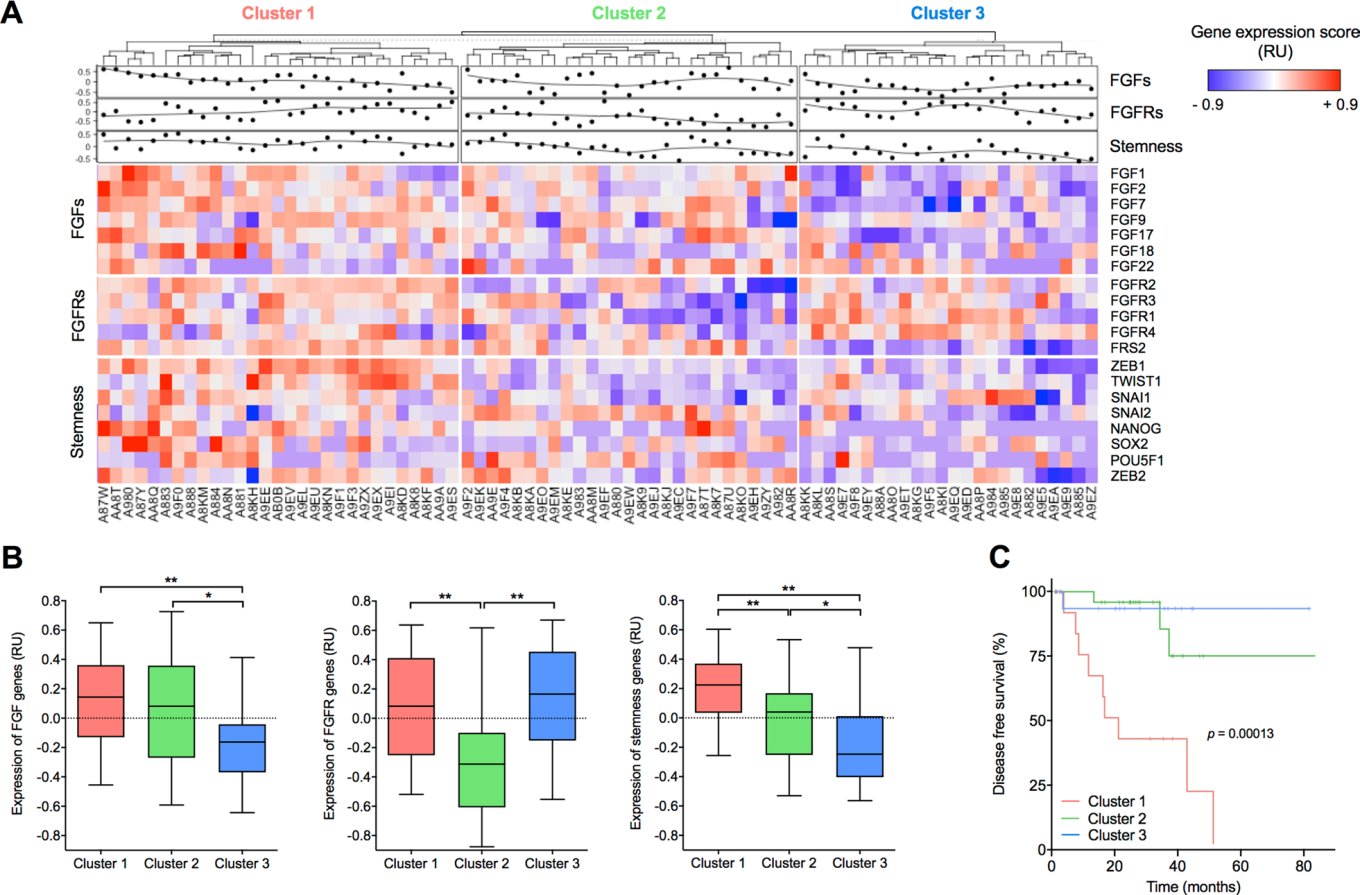
Hierarchical cluster analysis of gene expression data on TCGA UM Firehose Legacy Dataset. **A** Heatmap depicting the relative expression of the genes investigated on a cohort of 80 UM samples grouped by hierarchical cluster analysis. Each column represents one UM sample, and each row represents the indicated gene. The expression level of each gene in a single sample is depicted according to the color scale. **B** Fold change of the relative expression of FGF, FGFR and stemness genes in Cluster 1 (n = 29), Cluster 2 (n = 24), and Cluster 3 (n = 27). In box and whisker graphs, boxes extend from the 25th to the 75th percentiles, lines indicate the median values, and whiskers indicate the range of values. *p < 0.05, **p < 0.01, ANOVA. **C** Kaplan–Meier curve displaying Disease-Free Survival (DFS) of patients belonging to Cluster 1, Cluster 2, or Cluster 3. Log-rank test *p*-value. Relative units, RU

The analysis of the publicly available mRNA profiling dataset of UM patients, collected in The Cancer Genome Atlas (TCGA), indicates that the upregulation of FGFs and FGFRs is associated with a poorer prognosis as well as with chromosome 3 monosomy and *BAP1* mutation, two distinct molecular signatures that identify specific subsets of UM patients [21]. As already reported for other types of tumors, the high frequency of metastatic spreading, tumor relapse and/or therapy failure in UM have been attributed to the presence of a CSC subpopulation [22–24]. Based on our experimental evidence, we performed data mining on TCGA UM Firehose Legacy dataset (https://www.cbioportal.org/study/summary?id=uvm_tcga) to investigate the expression of FGF/FGFR family members and of transcription factors associated with stemness (*i.e. NANOG*, *OCT4*, *SNAIL*, *SLUG*, *TWIST*, *SOX2*, *ZEB1*, and *ZEB2*) on a cohort of 80 primary human UM specimens. Hierarchical clustering of the gene expression data identified three distinct molecular clusters, hereinafter referred to as Cluster 1 (n = 29), Cluster 2 (n = 24) and Cluster 3 (n = 27) associated with different levels of *FGFs*, *FGFRs* and stemness genes (Fig. 5A). In detail, Cluster 1 comprised patients characterized by the upregulation of both *FGFs* and *FGFRs* (including the downstream FGFR-mediator *FRS2*) and was associated with the highest levels of stemness-related transcription factors. Conversely, Cluster 2 was defined by intermediate levels of *FGFs* and of stemness genes, with low expression of *FGFRs*. Finally, Cluster 3 was characterized by high levels of *FGFRs* and lower expression of both ligands and stemness-associated transcription factors (Fig. 5B). Together, these data point to a tight relationship between activation of the FGF/FGFR system and UM stemness in clinical settings. Notably, these three clusters were associated with a distinct Disease-Free Survival (DFS), with patients belonging to Cluster 1 showing the worst prognosis (*p* = 0.0001 vs Cluster 2 and Cluster 3) (Fig. 5C).

Altogether, these results indicate that FGF/FGFR expression and stemness are strictly linked in UM patients and their clustering identifies more aggressive tumors characterized by a poorer prognosis.

## Discussion

A major issue in the management of UM patients is represented by the ability of tumor cells to metastasize to distant organs. This event is the consequence, at least in part, of the presence of a UM-CSC subpopulation, characterized by the ability to initiate tumorigenesis and self-renewal. In the present study, we demonstrate that the UM-CSC subpopulation strongly depends on the FGF/FGFR signaling and that FGF-trapping represents a strategy to efficiently hamper the growth of UM cells in vitro and in vivo.

A variety of FGFs and FGFRs are overexpressed by a significant subset of primary eye cancers, particularly in retinoblastoma and UM [17]. However, information about the pleiotropic roles played by FGF/FGFR in ocular tumors is limited. From the clinical point of view, high levels of FGF2 found in UM primary specimens and liver metastasis are associated with an increased invasiveness and a worst prognosis [16]. Accordingly, activation of FGF/FGFR system has been identified as responsible for the resistance to bromodomain and histone deacetylase inhibitors [52]. In this context, blocking FGFs or their receptors resulted in reduced cell proliferation and survival in in vitro UM experimental models [14, 21]. Here, we expand these observations by showing that the blockade of FGF/FGFR axis suppresses the activation of a variety of intracellular phospho-kinases, it induces a strong mitochondrial oxidative stress response, and it inhibits tumor growth in the in vivo CAM model of tumor graft.

The fact that FGFs play a crucial role for the maintenance of stem cells has been reported both in physiological tissues as well as in a variety of tumor types. Indeed, the FGF/FGFR system is important during embryo development and both ligands and receptors are expressed by human embryonic stem cells, where they regulate proliferation and self-renewal [27, 29, 53, 54]. On the other hand, the FGF/FGFR system has recently been associated with the regulation of stem-like properties in CSCs, the subpopulation of tumor cells responsible for tumor maintenance, metastatic dissemination, chemoresistance, and tumor relapse [22]. For example, the activation of FGF-mediated signaling has been linked to therapy resistance and enrichment in CSCs in an experimental model of hepatocarcinoma [55]. Similarly, it has been reported that the FGF/FGFR system enhanced stemness by increasing stability and nuclear localization of SOX2 in pancreatic cancer [28], promoted the reversion of tumor cells to an undifferentiated, stem-like state in glioblastoma [56], and regulated CSCs through ERK signaling in a model of esophageal squamous cell carcinoma [57]. In this manuscript, we show that UM cells are a heterogeneous population which comprises stem-like cells, and we provide, for the first time, the rationale to select and target this cell population, by exploiting its higher sensitivity to the inhibition of FGF/FGFR system. Indeed, UM cells with a higher sensitivity to the FGF-trap NSC12 display several stem-like properties, such as increased expression of numerous stemness-related transcription factors, enhanced ALDH activity, and tumor-sphere formation capacity. Interestingly, in vivo “targeting” of this cell population results in the loss of the CSC subset, as well as in a reduction of tumor growth in the CAM model. These results are propaedeutic and set the basis for further investigation in more complex models. In addition, the analysis of UM patients’ database revealed that this strong association exists also at clinical level. In fact, the robust correlation between FGFs/FGFRs and stemness-related genes proves that the FGF/FGFR system represents a master regulator of cancer stemness also in primary UM tumors. Notably, the clinical outcome of these patients clearly shows that high expression of both ligands and receptors, as well as stemness-related transcription factors, is prognostic for a worse disease-free survival.

Recent experimental studies have demonstrated that UM-CSC eradication obviates to hepatic metastasization, thus pointing to UM-CSC as a potential therapeutic target [52]. Currently, several FGF/FGFR inhibitors are being evaluated in clinical trials for their efficacy on FGF-dependent tumors; however, their application is limited to tumors where the activity of the FGF/FGFR system is well described as a driver in tumor sustenance.

Based on our results, the clinical use of FGF/FGFR inhibitors, such as NSC12 or other FDA approved FGFRi, might be taken into consideration given their ability to target CSCs, which are known to be intrinsically resistant to conventional chemotherapy. Despite the necessity to perform additional studies, the possibility to exploit FGF/FGFR blockade in combination with other conventional/chemotherapy approaches should be assessed as a strategy to overcome drug resistance and recurrence in UM.

## Conclusions

In this paper, we demonstrated that sequestration of FGFs hits and unmasks a UM population with CSC properties. By targeting the UM-CSC subpopulation, blockade of FGFs inhibits UM growth both in vitro and in vivo. Moreover, we showed that FGF/FGF receptor expression and stemness are strictly linked in UM patients and are associated with poorer prognosis tumors. Altogether, these findings indicate that the FGF system plays a pivotal role in UM-CSC biology and may be exploited to develop novel anti-CSC strategies for UM.

## Supplementary Information

**Additional file 1: Fig S1.** Effect of FGF2 on the detachment phenotype induced by NSC12 on UM cells. 92.1 and Mel270 UM cells were treated with 15 μM NSC12 in the absence or in the presence of a 1:1 molar concentration ratio of FGF2. After 3 h, detached cells were collected and counted. **Fig S2.** Effect of NSC12 on paxillin phosphorylation. Densitometric analysis of immunoreactive band shown in Fig. 1D normalized to GAPDH protein levels. Data are the mean ± the SEM of two independent experiments. *p < 0.01 vs untreated, ANOVA. **Fig S3.** Formation of melanospheres and ALDH activity of UM cells. A) 3000 viable cells were resuspended in melanospheres culture medium and plated. After 7 days, melanospheres were counted. Data are the mean ± SEM of two independent experiments. B) ALDH^br^ br population was measured in 92.1, Mel270 and Mel285 cells by cytofluorimetric analysis according to manufacturer’s instructions. Data are the mean ± SEM of three independent experiments. C) Representative flow cytometry dot plots of Aldefluor + cells. The gate refers to the positive/negative cell populations as identified in the presence of DEAB inhibitor. **Fig S4.** Analysis of ALDH activity. Representative flow cytometry dot plots of Aldefluor+ cells of control or 7 μM NSC12 treated 92.1, Mel270 and Mel285 UM cells. The gate refers to the positive/negative cell populations as identified in the presence of DEAB inhibitor, according to manufacturer’s instructions. **Fig S5.** Effect of BGJ398 on the ALDH^br^ UM subpopulation. 92.1, Mel270 and Mel285 cells were treated with increasing doses of BGJ398 for 24 h. Then, ALDH^br^ cells were measured by cytofluorimetric analysis. Data are the mean ± SEM of three independent experiments. *p < 0.05 vs control, ANOVA. **Fig S6.** Effect of Dacarbazine on UM cells. A) UM cells were treated with increasing concentrations of Dacarbazine. After 72 h cells were counted. B) Mel285 and 92.1 cells were treated with increasing doses of Dacarbazine for 72 h. Then, ALDH^br^ cells were measured by cytofluorimetric analysis. Data are the mean ± SEM of two independent experiments. **Table SI.** Oligonucleotide primers used for semi-quantitative PCR analysis. Table SII. Oligonucleotide primers used for qPCR analysis.

## Data Availability

All data are included in the study and available from the corresponding author on reasonable request.
