# Supplementary material for: FGF-trapping hampers cancer stem-like cells in uveal melanoma
Source: Cancer Cell Int. 2023 May 11;23:89. doi: 10.1186/s12935-023-02903-z (PMC10173517; doi:10.1186/s12935-023-02903-z)
Supplement: Supplementary file 1 — Additional file 1: Fig S1. Effect of FGF2 on the detachment phenotype induced by NSC12 on UM cells. 92.1 and Mel270 UM cells were treated with 15 μM NSC12 in the absence or in the presence of a 1:1 molar concentration ratio of FGF2. After 3 h, detached cells were collected and counted. Fig S2. Effect of NSC12 on paxillin phosphorylation. Densitometric analysis of immunoreactive band shown in Fig. 1D normalized to GAPDH protein levels. Data are the mean ± the SEM of two independent experiments. *p < 0.01 vs untreated, ANOVA. Fig S3. Formation of melanospheres and ALDH activity of UM cells. A) 3000 viable cells were resuspended in melanospheres culture medium and plated. After 7 days, melanospheres were counted. Data are the mean ± SEM of two independent experiments. B) ALDHbr br population was measured in 92.1, Mel270 and Mel285 cells by cytofluorimetric analysis according to manufacturer’s instructions. Data are the mean ± SEM of three independent experiments. C) Representative flow cytometry dot plots of Aldefluor + cells. The gate refers to the positive/negative cell populations as identified in the presence of DEAB inhibitor. Fig S4. Analysis of ALDH activity. Representative flow cytometry dot plots of Aldefluor+ cells of control or 7 μM NSC12 treated 92.1, Mel270 and Mel285 UM cells. The gate refers to the positive/negative cell populations as identified in the presence of DEAB inhibitor, according to manufacturer’s instructions. Fig S5. Effect of BGJ398 on the ALDHbr UM subpopulation. 92.1, Mel270 and Mel285 cells were treated with increasing doses of BGJ398 for 24 h. Then, ALDHbr cells were measured by cytofluorimetric analysis. Data are the mean ± SEM of three independent experiments. *p < 0.05 vs control, ANOVA. Fig S6. Effect of Dacarbazine on UM cells. A) UM cells were treated with increasing concentrations of Dacarbazine. After 72 h cells were counted. B) Mel285 and 92.1 cells were treated with increasing doses of Dacarbazine for 72 h. Then, ALDHbr cells [file 12935_2023_2903_MOESM1_ESM.pdf]

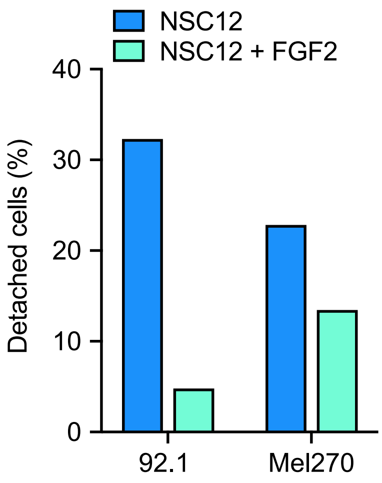


**Figure S1. Effect of FGF2 on the detachment phenotype induced by NSC12 on UM cells.** 92.1 and Mel270 UM cells were treated with 15 µM NSC12 in the absence or in the presence of a 1:1 molar concentration ratio of FGF2. After 3 h, detached cells were collected and counted.

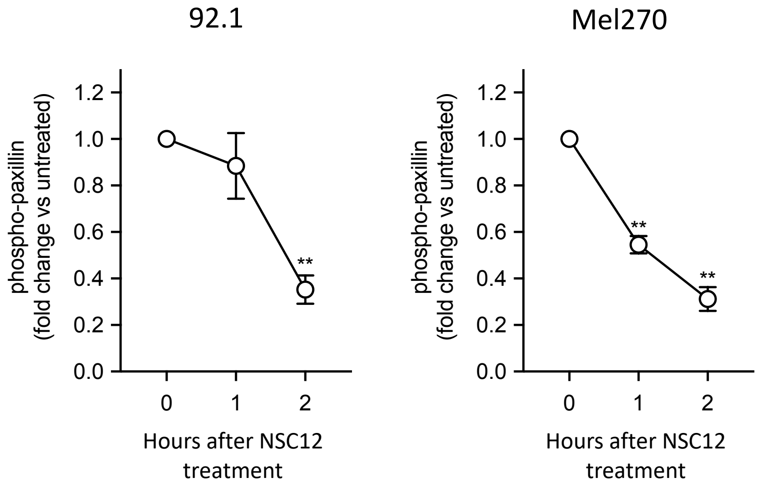


**Figure S2. Effect of NSC12 on paxillin phosphorylation.** Densitometric analysis of immunoreactive band shown in Fig. 1D normalized to GAPDH protein levels. Data are the mean ± the SEM of two independent experiments. **p* < 0.01 vs untreated, ANOVA.

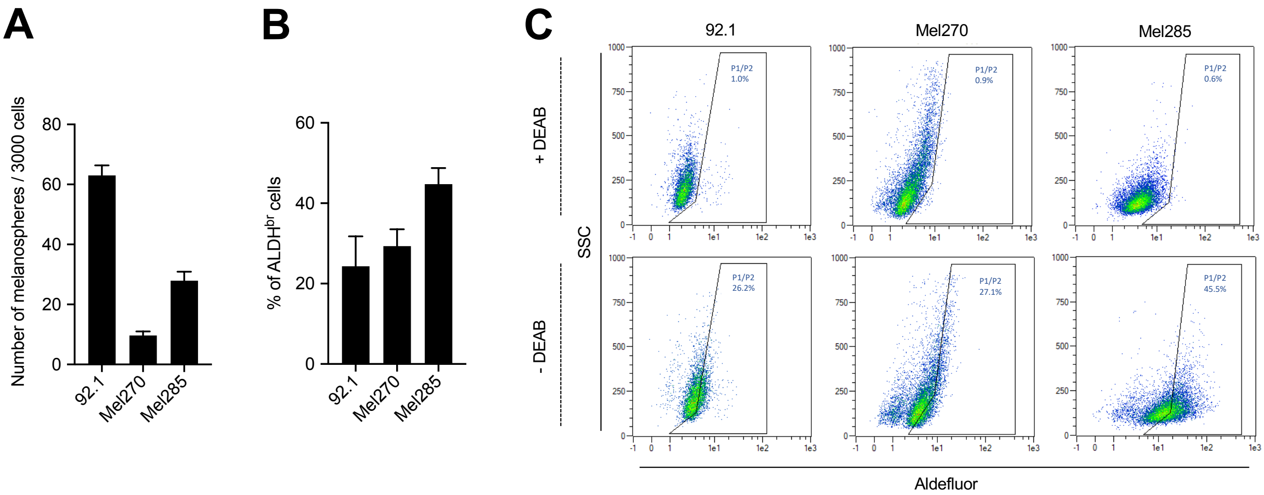


**Figure S3. Formation of melanospheres and ALDH activity of UM cells.** A) 3,000 viable cells were resuspended in melanospheres culture medium and plated. After 7 days, melanospheres were counted. Data are the mean ± SEM of two independent experiments. B) ALDH$^{br}$ population was measured in 92.1, Mel20 and Mel285 cells by cytofluorimetric analysis according to manufacturer's instructions. Data are the mean ± SEM of three independent experiments. C) Representative flow cytometry dot plots of Aldefluor+ cells. The gate refers to the positive/negative cell populations as identified in the presence of DEAB inhibitor.

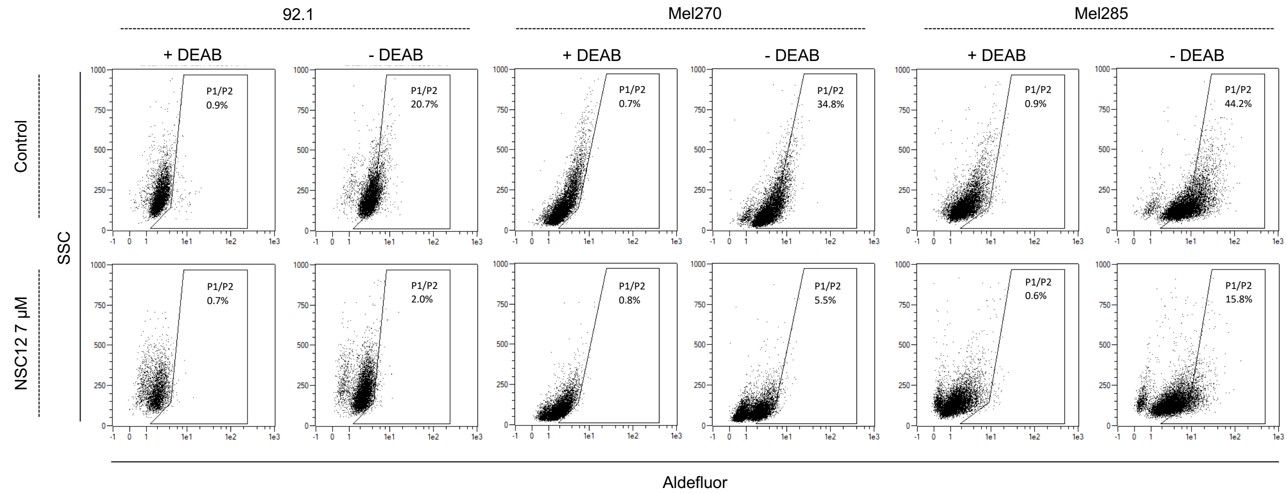


**Figure S4. Analysis of ALDH activity.** Representative flow cytometry dot plots of Aldefluor+ cells of control or 7 µM NSC12 treated 92.1, Mel270 and Mel285 UM cells. The gate refers to the positive/negative cell populations as identified in the presence of DEAB inhibitor, according to manufacturer's instructions.

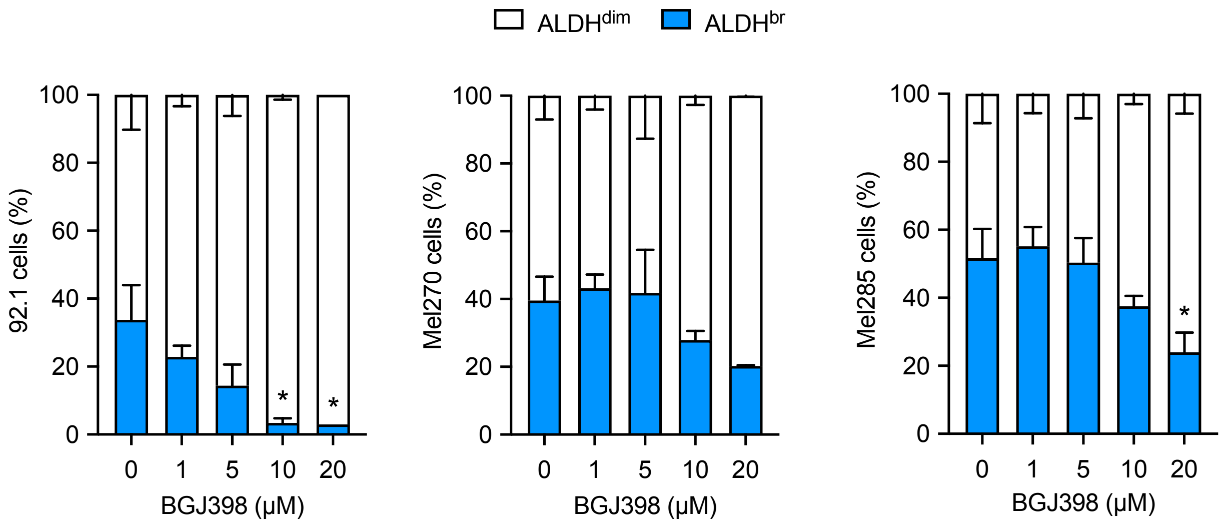


**Figure S5. Effect of BGJ398 on the ALDH$^{br}$ UM subpopulation.** 92.1, Mel270 and Mel85 cells were treated with increasing doses of BGJ398 for 24 h. Then, ALDH$^{br}$ cells were measured by cytofluorimetric analysis. Data are the mean ± SEM of three independent experiments. **p* < 0.05 vs control, ANOVA.

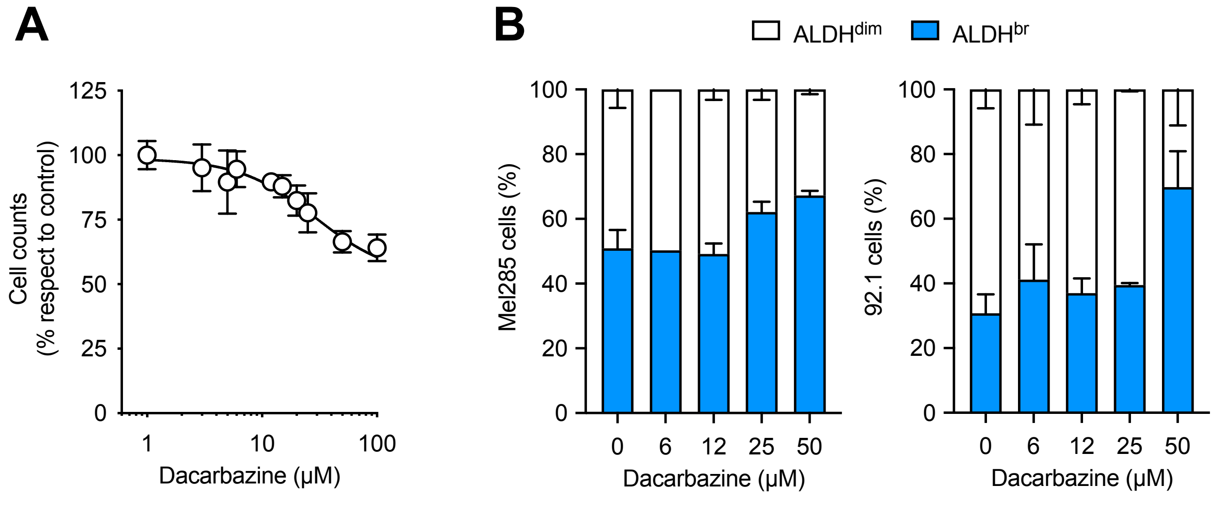


**Figure S6. Effect of Dacarbazine on UM cells.** A) UM cells were treated with increasing concentrations of Dacarbazine. After 72 h cells were counted. B) Mel285 and 92.1 cells were treated with increasing doses of Dacarbazine for72 h. Then, ALDH$^{br}$ cells were measured by cytofluorimetric analysis. Data are the mean ± SEM of two independent experiments.

**Supplementary Materials**

**Table S-I.** Oligonucleotide primers used for semi-quantitative PCR analysis.

| GENE | FORWARD PRIMER | REVERSE PRIMER |
|---|---|---|
| *FGF1* | *5’-TGAATGGGAACTCCCTTCC-3’* | *5’-GCTTTCAAGACTCTTTGCCT-3’* |
| *FGF2* | *5’-TGTGTCTATCAAAGGAGTGTG-3’* | *5’-CCGTAACACATTTAGAAGCCA-3’* |
| *FGF7* | *5’-TGACTTTGCCTCGTTTATCA-3’* | *5’-TGGCTACAAATGTGAACTGT-3’* |
| *FGF9* | *5’-CTGGATTTCACTTAGAAATCTTCCC-3’* | *5’-ATTCCAGAATGCCAAATCGG-3* |
| *FGF17* | *5’-CAACAAGTTTGCCAAGCTC-3’* | *5’-TACTTCTCACTCTCAGCCC-3’* |
| *FGF18* | *5’-GGACATGTGCAGGCTGGGCTA-3’* | *5’-GTAGAATTCCGTCTCCTTGCCCTT-3’* |
| *FGF22* | *5’-TCCACTCACTTCTTCCTGC-3’* | *5’-GATCTCCAGGATGCTGTCC-3’* |
| *FGFR1* | 5’-GGGCTGGAATACTGCTACAA-3’ | 5’-GCCAAAGTCTGCTATCTTCATC-3’ |
| *FGFR2* | 5’-GGATAACAACACGCCTCTCTT-3’ | 5’-GCCCAAAGCAACCTTCTC-3’ |
| *FGFR3* | 5’-TGGTGTCCTGTGCCTACC-3’ | 5’-CCGTTGGTCGTCTTCTTGT-3’ |
| *FGFR4* | 5’-AACCGCATTGGAGGCATT-3’ | 5’-TCTACCAGGCAGGTGTATGT-3’ |
| *GAPDH* | *5’-GAAGGTCGGAGTCAACGGATT-3’* | *5’-TGACGGTGCCATGGAATTTG-3’* |

**Table S-II.** Oligonucleotide primers used for qPCR analysis.

| GENE | FORWARD PRIMER | REVERSE PRIMER |
|---|---|---|
| *NANOG* | *5’-ACCTATGCCTGTGATTTGTGG-3’* | *5’-AAGTGGGTTGTTTGCCTTTG-3’* |
| *OCT4* | *5’-GCAAAGCAGAAACCCTCGT-3’* | *5’-ACACTCGGACCACATCCTTC-3’* |
| *TWIST* | *5’-GTCCGCAGTCTTACGAGGAG-3’* | *5’-GCTTGAGGGTCTGAATCTTGCT-3’* |
| *SOX2* | *5’-CATCACCCACAGCAAATGAC-3’* | *5’-CACCCTCCCCAGGTTTTC-3’* |
| *SNAIL* | *5’-AATCGGAAGCCTAACTACAGCG-3’* | *5’-GTCCCAGATGAGCATTGGCA-3’* |
| *SLUG* | *5’-TGACCTGTCTGCAAATGCTC-3’* | *5’-CAGACCCTGGTTGCTTCAA-3’* |
| *CD44* | *5’-AACACCAAGCCCAGAGGAC-3’* | *5’-TCCAAATCTTCCACCAAACC-3’* |
| *CD47* | *5’-TCCAAGAATGATGCCTTTCA-3’* | *5’-TACTGCCATAACTGCCCAAA-3’* |
| *GAPDH* | *5’-GAAGGTCGGAGTCAACGGATT-3’* | *5’-TGACGGTGCCATGGAATTTG-3’* |
